# MitraClip intervention for Sheehan syndrome complicated by dilated cardiomyopathy and valvular insufficiency: a case report and literature review

**DOI:** 10.3389/fendo.2025.1593741

**Published:** 2025-07-16

**Authors:** Liu Yan, He Wujin, Wang Hairong, Zhou Mei, Liao Farong, Yu Wei, Zhang Qi

**Affiliations:** ^1^ School of Nursing, Sun Yat-Sen University, Guangzhou, Guangdong, China; ^2^ Department of Nursing, Affiliated Hospital of Guilin Medical University, Guilin, Guangxi, China; ^3^ Ward One, Department of Cardiovascular Medicine, Affiliated Hospital of Guilin Medical University, Guilin, Guangxi, China

**Keywords:** mitraclip, Sheehan syndrome, dilated cardiomyopathy, valvular insufficiency, case report

## Abstract

**Introduction:**

Sheehan syndrome (SS) is a form of hypopituitarism caused by severe postpartum hemorrhage. It leads to premature dysfunction of the target organs affected by various hormone deficiencies, resulting in a range of clinical manifestations. The coexistence of SS and dilated cardiomyopathy is rare, and symptomatic treatment remains the main treatment approach. Here, we describe a case in which a MitraClip procedure was used to treat SS-associated cardiac insufficiency.

**Case report:**

A 50-year-old woman with a history of SS and dilated cardiomyopathy was admitted to the hospital owing to worsening shortness of breath for one month, which had aggravated over the past day. Her laboratory findings were as follows: potassium 5.92 mmol/L, chloride 96.56 mmol/L, magnesium 1.08 mmol/L, triiodothyronine 0.69 nmol/L, free thyrogenic ammonia 2.44 pmol/L, and B-type natriuretic peptide precursor 23,904 pg/mL. Medical imaging revealed left atrial and left ventricular enlargement (left atrial size 39 mm, left ventricular diameter 52 mm); severe regurgitation of the second and tricuspid valves; bilateral pleural effusion; abdominal, pelvic, and pericardial effusion Despite repeated pharmacological treatment, the patient’s condition did not improve. Finally, she underwent atrial septal puncture followed by percutaneous mitral valve repair using a MitraClip. The left Atrial diameter (LAS) decreased from 39mm (pre-intervention) to 34mm (pre-discharge), and decreased to 24mm during the 9-month outpatient follow-up. The left ventricular ejection fraction (LVEF) increased from 31% (pre-intervention) to 46% during the 9-month outpatient follow-up, and the pulmonary artery systolic pressure (PASP) decreased from 52 mmHg (pre-intervention) to 26 mmHg during the 9-month outpatient follow-up.

**Conclusion:**

The coexistence of Sheehan syndrome and dilated cardiomyopathy is rare. MitraClip intervention may be considered to treat severe mitral valve regurgitation due to dilated cardiomyopathy induced by Sheehan syndrome in cases where medical therapy is ineffective and the patient has significant symptoms of heart failure.

## Introduction

Sheehan syndrome (SS) is a form of hypopituitarism caused by severe postpartum hemorrhage (1). This disorder was first described in 1939 by Sheehan and Murdoch (2). It leads to the dysfunction of target organs owing to hormonal deficiencies, resulting in a range of clinical manifestations (1). Acute SS usually occurs within two weeks after delivery and presents with various initial symptoms, including adrenal hypocorticosis, diabetes insipidus, hypothyroidism, and panhypopituitarism. When promptly diagnosed, appropriate treatment can be initiated to prevent complications (3). However, dilated cardiomyopathy is a rare and delayed presentation of SS. Owing to its hidden onset and difficulty in early detection, it often leads to irreversible myocardial enlargement and valvular heart disease (4).

In addition, repeated hospitalizations are required, as symptoms cannot be adequately controlled with hormone therapy and symptomatic supportive treatment alone. Herein, we report a case in which a MitraClip procedure was used to treat SS-associated cardiac insufficiency. Additionally, we review the relevant literature to provide a reference for the diagnosis and treatment of similar cases.

## Case report

A 50-year-old woman was admitted to the hospital with shortness of breath persisting for one month, which had worsened over the past day. She had been diagnosed with SS 20 years ago following postpartum hemorrhage and had a history of hypothyroidism, hyperlipidemia, and fatty liver. Over the past month, she experienced shortness of breath and moderate symmetrical pitting edema in both lower limbs after physical activity. She had been previously diagnosed with dilated cardiomyopathy, valvular heart disease, and heart failure, for which she had received multiple treatments. On admission, her physical examination findings were as follows: body temperature 36.2°C, pulse 103 beats/min, respiratory rate 20 breaths/min, and blood pressure 102/70 mmHg.

The patient was conscious, anemic, and alert. There was no swelling of the tonsils, sclera, skin, or superficial lymph nodes. Auscultation revealed wet rales in both lungs. Cardiac examination showed an irregular rhythm, audible mitral valve sounds, and diastolic murmurs. The abdomen was distended but non-tender, with no rebound pain. The liver and spleen were not palpable below the costal margin, and there was no percussion pain in the liver, spleen, or kidney regions. Mobility dullness was negative, bowel sounds were normal, and moderate symmetrical pitting edema was present in both lower limbs.

Laboratory findings of the third on admission were as follows: potassium 5.92 mmol/L, chloride 96.56 mmol/L, magnesium 1.08 mmol/L, triiodothyronine 0.69 nmol/L, free thyroxine 2.44 pmol/L, and B-type natriuretic peptide precursor 23,904 pg/mL. The results of the patient’s three laboratory tests are summarized in **Table 1**.

**Table 1:** Normal ranges of laboratory tests and patient data across hospitalizations.

Laboratory Tests	Normal range/ Date of hospitalizations					
	2024-05-05 (admitted for the first time)	2024-05-20 (the second) on admission	2024-06-02 (the second discharge)	2024-06-04 (the third) on admission	2024-06-28 (the third discharge)	2024-11-08 (outpatient return visit)
Red blood cell count (×10¹²/L)	3.49	3.35	2.74	3.08	3.42	4.82
Hemoglobin concentration (g/L)	111.00	105.00	88.00	100.00	114.00	150.00
NT-proBNP (pg/mL)	6,982.00	15,883.00	14,727.00	23,904.00	5505.00	–
D-dimer(II)(µg/mL)	–	5.81	1.77	1.57	0.33	–
Direct bilirubin (µmol/L)	3.36	40.62	8.58	12.77	7.5	1.4
Total protein (g/L)	65.2	55.4	57.1	64.2	54.4	76.1
Alanine aminotransferase (U/L)	10.5	2595.4	207.6	202.5	56.8	30.1
Aspartate transferase (U/L)	31.00	5345.5	88.7	97.3	37.2	30.3
Venous blood glucose (mmol/L)	6.37	3.7	–	7.01	6.00	4.51
Urea nitrogen (mmol/L)	4.4	10.3	2.6	18.00	10.9	10.2
Creatinine (µmol/L)	95.00	161	66	131	76.00	73
Potassium ions (mmol/L)	3.73	3.76	4.51	5.92	4.3	4.12
Sodium ions (mmol/L)	134.23	124.85	140.8	136.12	134.9	139.4
Chloride ions (mmol/L)	97.5	95.00	102.86	96.56	102.3	100.5
Cortisol (4pm) (µg/dL)	12.42	>60.00	–	29.34	–	–
Cortisol (12pm) (µg/dL)	9.41	>60.00	–	21.41	–	–
Cortisol (8am) (µg/dL)	10.42	55.48	–	11.74	–	–
T4 Thyroxine (T4) (nmol/L)	–	47.9	–	107.00	96.9	150.00
Free thyroid hormone (FT4) (pmol/L)	3.29	7.28	–	16.2	21.6	21.3
Triiodothyronine (T3) (nmol/L)	–	0.44	–	0.69	0.56	1.27
Free thyrogenic ammonia (FT3)(pmol/L)	1.08	1.83	–	2.44	2.12	4.14
Thyroid-stimulating hormone (TSH) (uIU/mL)	2.18	0.67	–	2.64	1.54	0.56

**Figure 1:**
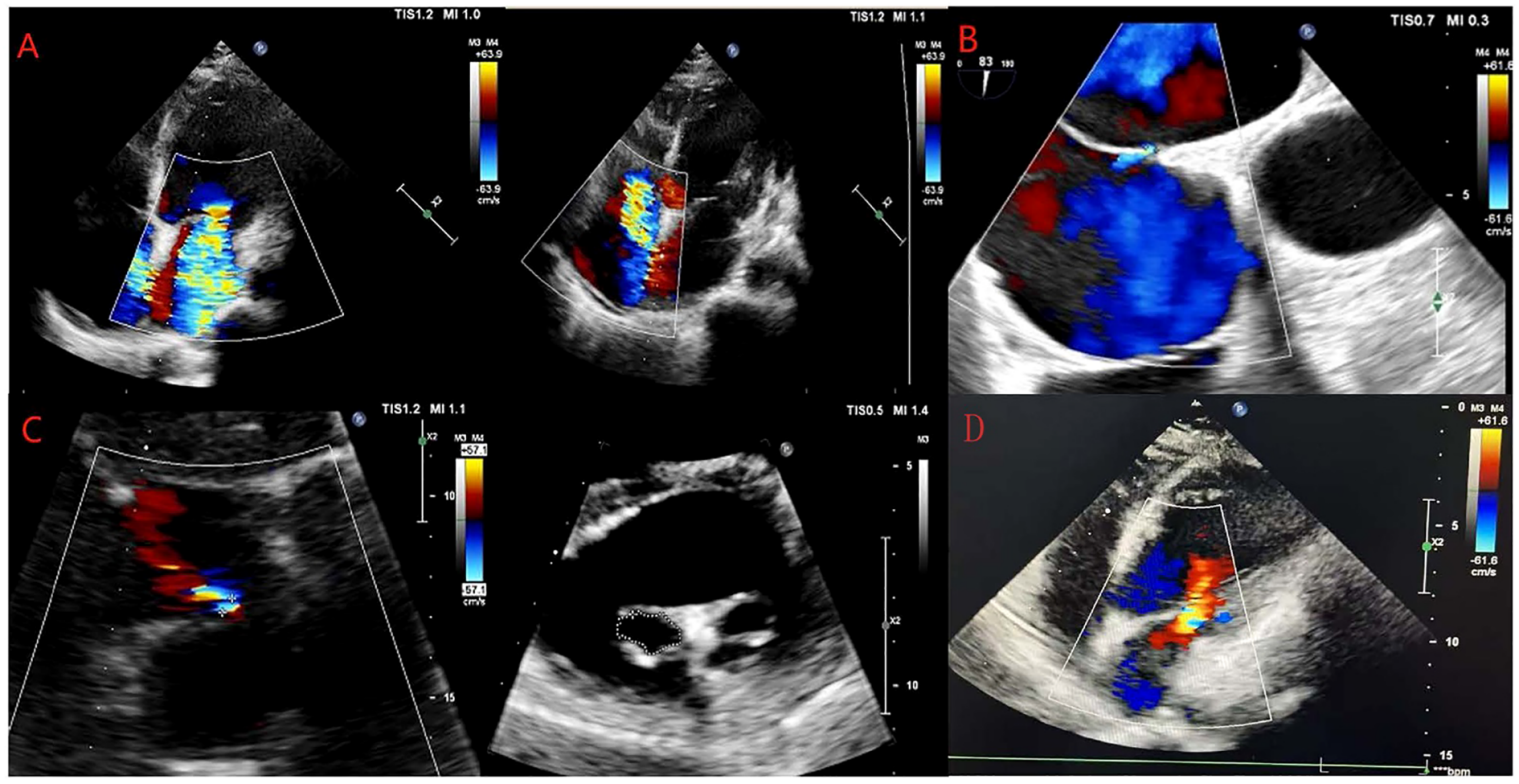
Changes in echocardiography examinations of the patient. **(A–D)** show the results of the echocardiography examinations of the patient on May 31, 2024, June 11, 2024, June 24, 2024, and February 14, 2025.

Medical imaging results revealed left atrial and left ventricular enlargement (left atrial size 39 mm, left ventricular diameter 52 mm); severe regurgitation of the second and tricuspid valves; bilateral pleural effusion; abdominal, pelvic, and pericardial effusion. The patient’s medical imaging findings are shown in **Figure 1**.

**Figure 2:**
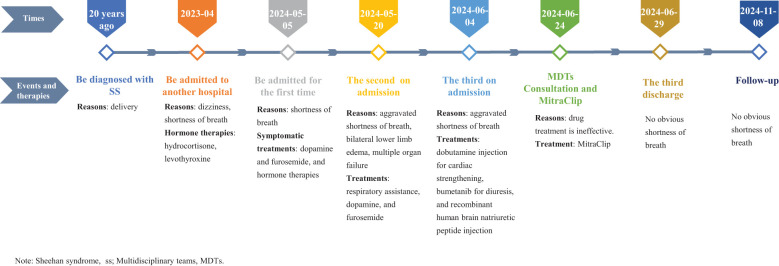
A timeline of the events. ss, Sheehan syndrome; MDTs, Multidisciplinary teams.

Upon admission, the patient was diagnosed with dilated cardiomyopathy with severe mitral valve insufficiency, moderate tricuspid valve insufficiency, acute exacerbation of chronic heart dysfunction (New York Heart Association, NYHA class IV), SS, arrhythmias with complete right bundle branch block, renal insufficiency, right pleural effusion, abdominal effusion, hyperacidemia, and abnormal liver function. She was treated with symptomatic supportive measures, such as anticoagulation, diuresis, gastric protection, correction of electrolyte disturbances, and hormone therapy. However, the patient was admitted to the hospital due to repeated shortness of breath. After about one year of hormone treatment with levothyroxine and hydrocortisone, about two months of diuretic treatment with furosemide or bumetanib, and dopamine-based cardiotonic drugs, the patients’ symptoms still could not be well relieved. After multidisciplinary team (MDTs) consultation, it was considered that repeated pharmacological treatment was ineffective, and MitraClip was ultimately chosen based on the patient’s symptoms and the results of echocardiography. This timeline of the events was shown in **Figure 2**. Following a comprehensive evaluation, the patient underwent atrial septal puncture and percutaneous mitral valve repair on June 24, 2024.

In this case, one clip was implanted in the patient. The MitraClip intervention process was mainly carried out in the cardiac catheterization laboratory. This includes inserting the guide tube through the right femoral vein into the atrium, locating the guide tube using transesophageal echocardiography and X-ray fluoroscopy images, sending the guide tube into the MitraClip forming clip delivery system through the guide tube, opening the MitraClip forming clip, and further adjusting its position. Then it was confirmed that the two arms of the MitraClip clip fell on the two leaflets of the left ventricle, the mitral regurgitation was significantly reduced, and the trans-valve pressure difference of the mitral valve was < 5 mm Hg. Finally, the MitraClip clip was released, the MitraClip clip delivery system and guide tube were removed, and the femoral vein and artery were closed by the vascular closure device.

## Follow-up and outcomes

Postoperative follow-up cardiac ultrasound revealed mild mitral valve lobe regurgitation and improved heart function. The echocardiographic values of the patients underwent long-term and significant changes from pre-intervention to post-intervention. For example, Left Atrial diameter (LAS) decreased from 39mm (pre-intervention) to 34mm (pre-discharge), and decreased to 24mm during the 9-month outpatient follow-up. The left ventricular ejection fraction (LVEF) increased from 31% (pre-intervention) to 46% during the 9-month outpatient follow-up, and the pulmonary artery systolic pressure (PASP) decreased from 52 mmHg (pre-intervention) to 26 mmHg during the 9-month outpatient follow-up. The patient’s echocardiographic parameter changes are shown in **Table 2**. The laboratory reexamination results are shown in **Table 1**. The patient experienced no significant discomfort, and her overall condition improved. She was discharged on June 29, 2024, and underwent a follow-up evaluation at the outpatient clinic on November 8, 2024, and February 14, 2025. During the outpatient follow-up of the patients, the NYHA classification and the 6-minute walk test (6MWT) were evaluated. On July 21st, the 6MWT was 253m, which was NYHA class II. On November 8th, the 6MWT was 350m, and the cardiac function was NYHA class II. On February 14, 2025, the 6MWT was 515m, and the cardiac function was NYHA class II.

**Table 2:** Sheehan’s syndrome with echocardiographic parameter changes.

Categories	2024-05-05 (admitted for the first time)	2024-05-31 (pre-intervention)	2024-06-26 (pre-discharge)	2024-07-12 (outpatient visit 1)	2024-11-08 (outpatient visit 2)	2025-02-14 (outpatient visit 3)
LAS	38mm	39mm	34mm	31mm	30mm	24mm
RAS	31mm	35mm	24mm	33mm	24mm	26mm
LVDD	51mm	52mm	55mm	52mm	51mm	48mm
LVSD	43mm	44mm	49mm	47mm	40mm	37mm
RVD	21mm	23mm	19mm	23mm	21mm	19mm
LVEF	31%	31%	25%	21%	42%	46%
PASP	38mmHg	52mmHg	32mmHg	51mmHg	16mmHg	26mmHg

LAS, Left Atrial diameter; RAS, Right atrial diameter; LVDD, Left ventricular end-diastolic diameter; LVSD, Left ventricular end-systolic diameter; RVD, Right ventricular diameter; LVEF, Left ventricular ejection fraction; PASP, Pulmonary artery systolic pressure.

## Discussion

With advancements in medical care, the incidence of SS has decreased. However, the improved survival rate of affected women has led to the emergence of long-term complications, with dilated cardiomyopathy being one of the most common. The exact mechanism underlying dilated cardiomyopathy in SS remains unclear.

Previous studies have suggested that cardiomyopathy after secondary adrenocortical hypofunction is mainly due to insufficient secretion of adrenocortical hormone (especially cortisol) caused by pituitary dysfunction, which not only affects the metabolic and electrophysiological functions of the heart but also disrupts water and electrolyte metabolism, contributes to hypotension, increases cardiac workload, and triggers inflammatory responses. These factors collectively increase the risk of dilated cardiomyopathy and contribute to the development of heart failure (5, 6).

Most patients with SS complicated by dilated cardiomyopathy are hospitalized for many years, with durations ranging from 2 to 24 years after delivery (**Table 3**). They primarily present with cardiac symptoms, such as dyspnea, respiratory distress, and bilateral lower-extremity edema (3, 7–13). These symptoms often make accurate differential diagnosis and therapeutic recovery challenging. Currently, the treatment for SS complicated by dilated cardiomyopathy mainly focuses on hormone replacement therapy, which requires long-term administration. However, in patients with multiple comorbidities, symptoms can be difficult to control, leading to frequent hospital visits and imposing significant psychological and economic burdens on both patients and their families. Therefore, identifying effective and timely strategies to alleviate the symptom burden of patients remains important.

**Table 3:** Sheehan’s syndrome with reversible dilated cardiomyopathy: treatment, and therapeutic response.

Authors, years, country	Number of patients	Onset time	Diagnosis	Treatment	Response	Reference number
Islam AK (2014, Dhaka)	1	2 years after delivery	Sheehan syndrome with reversible dilated cardiomyopathy	diuretics, ACE inhibitors, digoxin, and hydrocortisone, prednisolone.	The patient’s breathlessness decreased, edema disappeared, and psychological status improved. The reduction of chamber dimensions improved myocardial contractility, MR disappeared, and, systolic dysfunction was completely recovered.	(8)
Makharia A (2021, India)	1	24 years after delivery	Sheehan syndrome with recurrent hypoglycemia and dilated cardiomyopathy	intravenous dextrose solution, intravenous hydrocortisone, prednisolone, thyroxine supplementation, spironolactone, ACE inhibitors, beta-blockers in low doses, digoxin, calcium and vitamin D.	A reduction in cardiac chamber dimensions and improvement in myocardial contractility was observed at 6 months on a repeat echocardiographic examination with the abovementioned treatment.	(3)
Dourado MLBF (2021, Brasil)	1	8 years after delivery	Sheehan syndrome with reversible dilated cardiomyopathy	intravenous hydrocortisone, prednisolone, levothyroxine	Reversal after 6 months of hormone replacement therapy	(9)
Laway BA (2010, India)	1	2 years after delivery	Sheehan syndrome with reversible dilated cardiomyopathy	antitubercular treatment, hydrocortisone, levothyroxine	Echocardiography revealed marked improvement at 4 and 7 months and at 7 months of follow up there was complete resolution of cardiac abnormalities.	(7)
Doshi S (2013, India)	1	14 years after delivery	Sheehan syndrome with dilated cardiomyopathy	glucocorticoids, thyroxine, and fludrocortisone	The patient was asymptomatic with normal chest radiograph and normal left-ventricular ejection fraction at 6 months follow-up.	(10)
Ikegami, Y (2016, Japan)	1	8 years after delivery	secondary adrenal insufficiency caused by Sheehan syndrome with idiopathic cardiomyopathy	oral cortisol therapy	Complete recovery of ejection fraction was noted, and the B-type natriuretic peptide level improved after 2 months of treatment. The cardiac function gradually improved follow-up with 1.5 years.	(11)
Hayat Bhat (2017, India)	1	10 days after delivery	Sheehan syndrome with reversible cardiomyopathy	glucocorticoids and levothyroxine	The blood counts and biochemistry showed significant improvement and got normalized. Echo cardiography after 3 months revealed normal LV systolic functions with EF=70% and complete resolution of hypokinesia.	(12)
Cheng Wang (2022, China)	1	2 years after delivery	Sheehan syndrome with cardiac insufficiency	diuretics, intravenous hydrocortisone infusion, and oral thyroxine	Cortisol levels and blood sodium levels were up to normal.	(13)

MitraClip intervention’s advantages over traditional surgery are less trauma and faster recorvery. Mitral valve clipping is a minimally invasive cardiovascular procedure primarily used to alleviate left atrial hypertension and heart failure symptoms caused by mitral regurgitation, which is common in SS accompanied by dilated cardiomyopathy and valve abnormalities (14). When varying degrees of mitral regurgitation are present, this procedure helps restore valve function by reducing the mobility of the mitral valve, thus decreasing the left atrial burden and improving cardiac pumping efficiency (15). In this case, the patient’s symptoms of SS complicated by dilated cardiomyopathy showed significant improvement at the 6-month follow-up. However, the MitraClip procedure is rarely used in patients with secondary dilated cardiomyopathy complicated by valvular heart disease. Its long-term efficacy and underlying mechanisms require further investigation and long-term follow-up to optimize treatment strategies.

In conclusion, although uncommon, MitraClip intervention may be considered to treat severe mitral valve regurgitation due to dilated cardiomyopathy induced by Sheehan syndrome in cases where medical therapy is ineffective and the patient has significant symptoms of heart failure.

## References

[1] Kovacs K. Sheehan syndrome. Lancet. (2003) 361:520–2. doi: 10.1016/S0140-6736(03)12490-7, PMID: 12583962

[2] Sheehan HL Murdoch R. Postpartum necrosis of the anterior pituitary: Production of subsequent pregnancy. Lancet. (1939) 233:818–20. doi: 10.1016/S0140-6736(00)60421-X

[3] Makharia A Lakhotia M Tiwari V Gopal K. Recurrent hypoglycaemia and dilated cardiomyopathy: delayed presentation of Sheehan's syndrome. BMJ Case Rep. (2021) 14:e242747. doi: 10.1136/bcr-2021-242747, PMID: 34162619 PMC8230959

[4] Matsuzaki S Endo M Ueda Y Mimura K Kakigano A Egawa-Takata T. A case of acute Sheehan's syndrome and literature review: a rare but life-threatening complication of postpartum hemorrhage. BMC Pregnancy Childbirth. (2017) 17:188. doi: 10.1186/s12884-017-1380-y, PMID: 28615049 PMC5471854

[5] Abdallah N Mohamoud A Kearns A Abdallah M Linzer M. Relationships between adrenal insufficiency and cardiovascular outcomes in patients with atrial fibrillation, atrial flutter and paroxysmal supraventricular tachycardia. Curr Probl Cardiol. (2024) 49:102641. doi: 10.1016/j.cpcardiol.2024.102641, PMID: 38754754

[6] Abdallah N Mohamoud A Daher H Abdallah M Mehfooz A. Relationships between adrenal insufficiency and cardiovascular outcomes in patients with congestive heart failure. Nutr Metab Cardiovasc Dis. (2024) 35(4):103835. doi: 10.1016/j.numecd.2024.103835, PMID: 39800620

[7] Laway BA Alai MS Gojwari T Ganie MA Zargar AH. Sheehan syndrome with reversible dilated cardiomyopathy. Ann Saudi Med. (2010) 30:321–4. doi: 10.4103/0256-4947.65269, PMID: 20622352 PMC2931786

[8] Islam AK Hasnat MA Doza F Jesmin H. Sheehan's syndrome with reversible dilated cardiomyopathy: A case report and brief overview. J Saudi Heart Assoc. (2014) 26:117–20. doi: 10.1016/j.jsha.2014.01.005, PMID: 24719543 PMC3978866

[9] Dourado M Costa TPD Carvalho MS Moura CGG. Dilated cardiomyopathy reversibility in Sheehan's syndrome: A case report. Arq Bras Cardiol. (2021) 116:17–20. doi: 10.36660/abc.20190547, PMID: 33566997 PMC8118631

[10] Doshi S Roy A Ramamoorthy A Kothari SS Bahl VK. Dilated cardiomyopathy: a ghost from the past. Circ Heart Fail. (2013) 6:e19–21. doi: 10.1161/CIRCHEARTFAILURE.112.000062, PMID: 23513050

[11] Ikegami Y Fukuda T Jo R Momiyama Y. Reversible cardiomyopathy accompanied by secondary adrenal insufficiency. Circ Heart Fail. (2016) 9:e002919. doi: 10.1161/CIRCHEARTFAILURE.115.002919, PMID: 26920218

[12] Bhat MH Bagdadi F Rafi A Shah PA. Reversible cardiomyopathy as a rare presentation of sheehan’s syndrome-case report and review of literature. Int J Adv Med. (2017) 4:1713–5. doi: 10.18203/2349-3933.ijam20175199

[13] Cheng W Xiang C. Data from: A case of Sheehan syndrome complicated with cardiac insufficiency [J/OL. China Clin Case Repository. (2022).

[14] Heidenreich PA Bozkurt B Aguilar D Allen LA Byun JJ Colvin MM. 2022 AHA/ACC/HFSA guideline for the management of heart failure: A report of the American College of Cardiology/American Heart Association Joint Committee on Clinical Practice Guidelines. Circulation. (2022) 145:e895–e1032. doi: 10.1161/CIR.0000000000001063, PMID: 35363499

[15] Ailawadi G Lim DS Mack MJ Trento A Kar S Grayburn PA. One-year outcomes after MitraClip for functional mitral regurgitation. Circulation. (2019) 139:37–47. doi: 10.1161/CIRCULATIONAHA.117.031733, PMID: 30586701

